# IL-17-neutralizing antibody mitigates functional and structural changes in cigarette smoke-induced COPD model

**DOI:** 10.3389/fimmu.2025.1641300

**Published:** 2025-08-15

**Authors:** Alyne Riani Moreira, Camila Uchoa da Silva, Leticia de Paula Costa Mattos, Jussara Jesus Simão, Veronica Camargo Berti, Luan Henrique Vasconcelos Alves, Alex Ferreira da Silva, Franciele Jesus Lima, Suellen Karoline Moreira Bezerra, Cintia Nascimento Silva, Maria Isabel Cardoso Allonso- Vale, Iolanda de Fatima Lopes Calvo Tiberio, Francine Maria Almeida, Fernanda Degobbi Tenorio Quirino dos Santos Lopes

**Affiliations:** ^1^ Laboratory of Experimental Therapeutic (LIM 20), Division of Medicine – School of Medicine of Hospital das Clinicas HCFMUSP, Sao Paulo, Sao Paulo, SP, Brazil; ^2^ Graduate Program in Chemical Biology, Department of Biological Sciences, Federal University of São Paulo, São Paulo, SP, Brazil; ^3^ Thoracic Surgery Research Laboratory (LIM61), Division of Thoracic Surgery, Instituto do Coracao do Hospital das Clínicas da Faculdade de Medicina da Universidade de São Paulo, SP, Brazil

**Keywords:** COPD, adaptive immune response, structural changes, respiratory mechanics, IL-17 inhibitor

## Abstract

Smoking remains the main risk factor for the development of chronic obstructive pulmonary disease (COPD). The inflammatory response mediated by innate and adaptive immune cells has been described in the development and progression of the disease, and the importance of Th17 cytokines has been observed. Studies have shown that blocking interleukin (IL)-17 can reduce inflammation in experimental models of lung injury. This study evaluated the effect of an IL-17 inhibitor in a cigarette smoke-induced COPD model in C57BL/6 mice. The effects of treatment with an IL-17 inhibitor were evaluated in an experimental model of COPD. Mice were exposed to cigarette smoke for 6 months, and treatment with IL-17 inhibitor was initiated in the fifth month. Four experimental groups were constituted: Control group—animals housed in a vivarium, receiving filtered room air; Control anti-IL-17 group—animals housed in a vivarium, receiving filtered room air and treatment with an anti-IL-17-neutralizing antibody; COPD group—animals exposed to cigarette smoke; and COPD anti-IL-17 group—animals exposed to cigarette smoke and treated with an anti-IL-17-neutralizing antibody. In the COPD groups, an increase in mean linear intercept was observed, along with a decrease in tissue elastance and tissue damping, confirming the COPD development. Administration of the IL-17-neutralizing antibody reversed these structural and functional alterations. Additionally, the COPD group exhibited an inflammatory response characterized by increased infiltration of polymorphonuclear and mononuclear cells and elevated numbers of IL-17- and IL-6-positive cells. These findings were consistent with the increased expression of IL-17 and IL-6 in lung homogenates, as assessed by ELISA. Treatment with the IL-17-neutralizing antibody effectively reversed this inflammatory response by reducing the expression of these inflammatory markers. These results were further supported by the evaluation of *RORγt* gene expression, which was significantly upregulated in the COPD group. Treatment with the IL-17-neutralizing antibody alleviates this upregulation. Thus, this study demonstrates, for the first time, the effectiveness of IL-17-neutralizing antibody treatment in a cigarette smoke-induced model of COPD, even after lung damage had been established, suggesting the therapeutic potential of IL-17-neutralizing antibodies in COPD.

## Introduction

Chronic obstructive pulmonary disease is a heterogeneous lung disease characterized by chronic respiratory symptoms, such as dyspnea, cough, sputum production, and exacerbation, due to abnormalities of the airways (bronchitis, bronchiolitis) and/or alveoli (emphysema), causing persistent, often progressive, airflow obstruction. Smoking remains a main risk factor for the development and progression of chronic obstructive pulmonary disease (COPD) (1–5).

Although there are many current therapeutic approaches to managing COPD, healthcare professionals face numerous challenges in this field. These challenges can be attributed to the heterogeneity of the pathophysiological mechanisms involved in the development and progression of COPD (6). In COPD patients with frequent exacerbations and elevated blood eosinophil levels, the addition of inhaled corticosteroids to the double bronchodilator regimen should be considered (1, 7). However, the use of steroids is associated with many adverse events, mainly related to the cumulative dose due to common recurrent pulmonary infections in COPD patients (8).

The inflammatory response mediated by innate and adaptive immune cells has been described in the development and progression of COPD, and the importance of Th17 cytokines has been observed since the early stages of this disease (9, 10). Neutrophil recruitment is present from the early stages and is mainly associated with interleukin (IL)-17A and IL-17A/F activation. Activation of IL-17 receptors expressed on bronchial epithelial cells orchestrates the secretion of neutrophil chemotactic factors such as granulocyte colony-stimulating factor or the chemokine CXC ligand (CXCL)8, cytokines such as IL-6, and antimicrobial peptides such as β-defensins and S100 proteins (11–13).

Regarding the adaptive immune response, depending on the COPD stage, Th17 cytokines can be found in lung and blood samples. Lourenço et al. (14) evaluated the gene expression of intracellular proteins and related cytokines involved in Th17 response in both mild and moderate COPD patients and compared local and systemic responses. They showed that intracellular signaling for the Th17 response is also present from the early stages of the disease. Th17 markers were observed in lung samples from patients with mild COPD, whereas in moderate stages, they were also detected in blood samples.

The importance of steroid therapy in type 17-mediated pulmonary inflammation has already been studied in other pulmonary disease models (15, 16). Additionally, previous studies have shown that anti-IL-17 administration decreases inflammation in experimental models of lipopolysaccharide (LPS)-induced acute lung injury and chronic allergic lung inflammation exacerbated with LPS (17, 18). Moreover, Fukuzaki et al. (19) investigated the therapeutic effects of intraperitoneal anti-IL-17 administration in an elastase-induced lung injury model in mice, which requires a relatively short time to induce emphysematous characteristics. They observed a reduction in most functional impairments, inflammatory mediators, and lung remodeling parameters associated with this type of lung injury. Considering that tobacco smoking is the main etiological factor contributing to the development of COPD in humans, our research group previously investigated the importance of Th17 cytokines in an animal model of cigarette smoke (CS) exposure and a CS-induced model associated with LPS administration. We showed that, in both models, there was a skewing toward a Th17 immune response, which was associated with a decline in respiratory mechanics and changes in histological parameters that characterize COPD development, resembling certain clinical findings in humans.

Thus, in this study, we evaluated the effects of administering an IL-17-neutralizing antibody on functional and histological lung parameters in a CS-induced COPD model in mice.

## Methods

### Animals

The present study was approved by the Ethics Committee on Human and Animal Research of the Faculty of Medicine, University of São Paulo (Animal Use Ethics Committee – CEUA protocol number 1452/2020). Male C57BL/6 mice, 6–8 weeks old, were used. All animals received humane care in accordance with the National Institutes of Health Guide for the Care and Use of Laboratory Animals (NIH Publication No. 85-23, revised 1996).

### Induction of emphysema by cigarette smoke exposure

Mice were exposed to 24 commercially filtered cigarettes per day (Souza Cruz – BAT, Brazil; 10 mg of tar, 0.8 mg of nicotine, and 10 mg of CO per cigarette Av. José Andraus Gassani, 5002-5268 - Distrito Industrial, Uberlândia - MG, 38402-338) (20), A 30-min full-body exposure to CS from 12 cigarettes was performed twice a day for 5 days/week for 24 weeks (6 months). The exposure was conducted in an inhalation chamber, a 28-L plastic box (approximately 40 cm × 27 cm at the base, with a height of 26 cm) with two air inlets: synthetic air and cigarette smoke. A small fan for air homogenization is at the top of the box. The airflow inside the compartment is controlled by a flow meter connected to a compressed air torpedo and is maintained at 2 L/min. The second air inlet receives a mixture of synthetic air and cigarette smoke, drawn in by a Venturi system connected to the lit cigarette. The laminar flow of synthetic air passes through a region of smaller diameter, resulting in an acceleration of the flow and a consequent reduction in pressure at this point, known as the Venturi effect, which facilitates the aspiration of cigarette smoke. The decrease in pressure at the point of diameter reduction in the tube depends on the airflow, which is kept constant. This system creates a concentration of carbon monoxide ranging from 250 to 350 ppm (ToxiPro, Biosystems, USA, 6616 Owens Drive, Pleasanton, Califórnia, EUA) (21).

### IL-17-neutralizing antibody administration

An anti-IL-17A-neutralizing antibody (clone 50104; R&D Systems, Abingdon, UK) was administered by intraperitoneal injection 1 h before each cigarette smoke exposure (2 × per week) at a dose of 7.5 μg/application during the last month of exposure. Each animal received eight applications in total (18, 22).

### Experimental groups

Animals were randomly divided into four groups:

*Control (CONTROL):* animals maintained under filtered air (*n* = 7).

*Control anti-IL-17 group (CONTROL anti-IL-17):* animals maintained under filtered air that received IL-17-neutralizing antibody administration (*n* = 7).

*COPD group (chronic obstructive pulmonary disease [COPD]):* animals exposed to cigarette smoke for 6 months (*n* = 8).

*COPD group + anti-IL-17 (COPD anti-IL-17):* animals were exposed to cigarette smoke for 6 months and received IL-17-neutralizing antibody administration (*n* = 8).

Two experimental setups were necessary for the protocol. In the first, lungs (right and left) were used to evaluate respiratory mechanics parameters and conduct morphometric analysis. The second setup was required to obtain lung homogenates for cytokine and gene expression analysis.

### Respiratory mechanics

At the final step of the CS exposure protocol, animals were anesthetized with thiopental (50 mg/kg, intraperitoneally), tracheostomized, and placed on a rodent mechanical ventilator (flexiVent, SCIREQ, Montreal, Canada) for respiratory mechanics assessment. They were ventilated with a tidal volume of 10 mL/kg and a respiratory rate of 120 cycles/min. Using the previously described constant-phase model, airway resistance (Raw), tissue damping (Gtis), and tissue elastance (Htis) parameters were calculated (23).

### Lung preparation

After the respiratory mechanic assessment, mice were submitted to euthanasia by exsanguination through the abdominal aorta under anesthesia, and their entire lungs were removed and fixed at a constant pressure of 20 cmH_2_O using 10% buffered formalin infused through the trachea for 24 h (15). The lungs were embedded in paraffin and cut into 5-µm sections for histological and morphometric evaluation.

### Morphometry

The tissue samples were stained with hematoxylin and eosin (H&E) for conventional morphometry to perform the mean linear intercept (Lm) measurements. Lm was obtained by counting the number of times that the lines of the reticulum, containing 50 lines and 100 points, intercepted the alveolar walls. We performed the Lm analysis in distal areas of the parenchyma (peripheral airspaces) and used the following equation: Lm = Ltotal/NI^6^, where Ltotal is the sum of all grid segments, calculated by measuring each segment with a ruler (Carl Zeiss Microscopy GmbH, Jena, Germany) attached to the microscope, and NI is the average number of times that the lines intersected the alveolar walls. All Lm values were expressed in micrometers (μm) (24).

### Cell density

To count the number of polymorphonuclear and mononuclear cells in the peribronchovascular areas, a Weibel reticle (100 points and 50 lines) was attached to the eyepiece of a standard optical microscope and used in a known area with the × 100 objective and immersion oil. The quantification of cells in the edema area between the vessel and the airway was done by reading 15 fields/slide (hematoxylin and eosin) (25).

### Positive cells by detected by immunohistochemistry for IL-17

Lung sections (5 µm thick) were deparaffinized and hydrated. Antigen retrieval was performed, and the sections were washed in phosphate-buffered saline (PBS) and blocked with 3% hydrogen peroxide at room temperature. The sections were then incubated with a mouse anti-IL-17 antibody (Santa Cruz, SC374218 10410 Finnell St, Dallas, TX 75220, Estados Unidos) at a dilution of 1:200. The primary antibody was diluted in bovine serum albumin (BSA) and incubated overnight (16–18 h) in a humidified chamber at 4°C–8°C. Subsequently, the sections were washed in PBS and incubated with a secondary antibody (Vector ABCElite, horseradish peroxidase [HRP]; antimouse) at 37°C in a humidified chamber. Three additional 5-min washes in PBS were performed, and the samples were stained with 3,3-diaminobenzidine (DAB) (code K3468, Dako Citomation, Fort Collins, CO, USA) for 5 min. Subsequently, the tissues were washed with tap water and counterstained with Harris hematoxylin. Cell density was assessed by the number of cells divided by the respective peribronchovascular area (10^4^ cells/µm^2^) in 15 fields/slide. Analysis was performed using an optical microscope equipped with an integrating eyepiece containing a known area (104 μm^2^ at × 1,000 magnification), consisting of 50 lines and 100 points (25).

### Cytokine analysis

The lungs were removed, stored in labeled tubes containing crushed ice, and then individually homogenized. Cytokine levels were detected by enzyme-linked immunosorbent assay (ELISA) (OptEIA, BD PharMingen, Oxford, UK) using microplates (Costar, Cambridge, MA, USA) sensitized with specific monoclonal antibodies for each cytokine. After the samples were washed, biotin-conjugated specific antibodies for the different cytokines were added. Next, a solution containing streptavidin–peroxidase, substrate, and chromogen enzyme conjugate was added. The reaction was read at 450 nm using an M2 spectrophotometer (Molecules Devices, San Jose, CA, USA). Sample concentrations were calculated from standard curves obtained with recombinant cytokines, and the results were expressed in picograms per milliliter. Interleukin (IL-6 and IL-17) and transforming growth factor beta (TGF-β) expression were determined using ELISA kits from R&D Systems (26).

### Real-time PCR

Gene expression was evaluated using real-time polymerase chain reaction (PCR) in a Rotor-Gene thermal cycler (Qiagen, Valencia, CA, USA) with a SYBR Green kit as a fluorescent marker (Qiagen, Valencia, CA, USA). The reactions occurred as follows: 95°C for 5 min; 40 cycles at 95°C for 5 s (denaturation) and 60°C for 10 s (annealing and extension). PCR products were run on an agarose gel to confirm fragment sizes and reaction specificity. Primers were designed and used to quantify messenger RNA (mRNA) encoded by the genes described below. Analysis was performed using the software provided by the manufacturer. Briefly, an arbitrary number of copies of the genes of interest and constitutive genes was calculated using the formula 1,000,000/2CT, where CT is the number of amplification cycles required to reach the threshold determined at the exponential phase of the curve for each sample. The values are presented as the number of copies relative to the control after correction with the constitutive gene *GAPDH* (26) (**Table 1**).

**Table 1:** Sense and antisense sequences of the primers used in qPCR.

Gene	5′ Primer (5′–3′)	3′ Primer (5′–3′)
*RORγt*	TAGCACTGACGGCCAACTTA	TCGGAAGGACTTGCAGACAT
*GAPDH*	CCACCACCCTGTTGCTGTAG	CTTGGGCTACACTGAGGACC

RORγt, Retinoic Acid Receptor-related Orphan Receptor gamma t.

### Statistical analysis

Statistical analysis was performed using the SigmaStat program (version 11.0; Systat Software, San Jose, CA, USA). A one-way ANOVA test was used, followed by the multiple-comparison test (Holm–Sidak test or Tukey test, depending on the normality of variables). Results were expressed as means ± standard error (SE), and a *p*-value less than 0.05 was considered statistically significant.

## Results

### Increase in Lm

**Figure 1:**
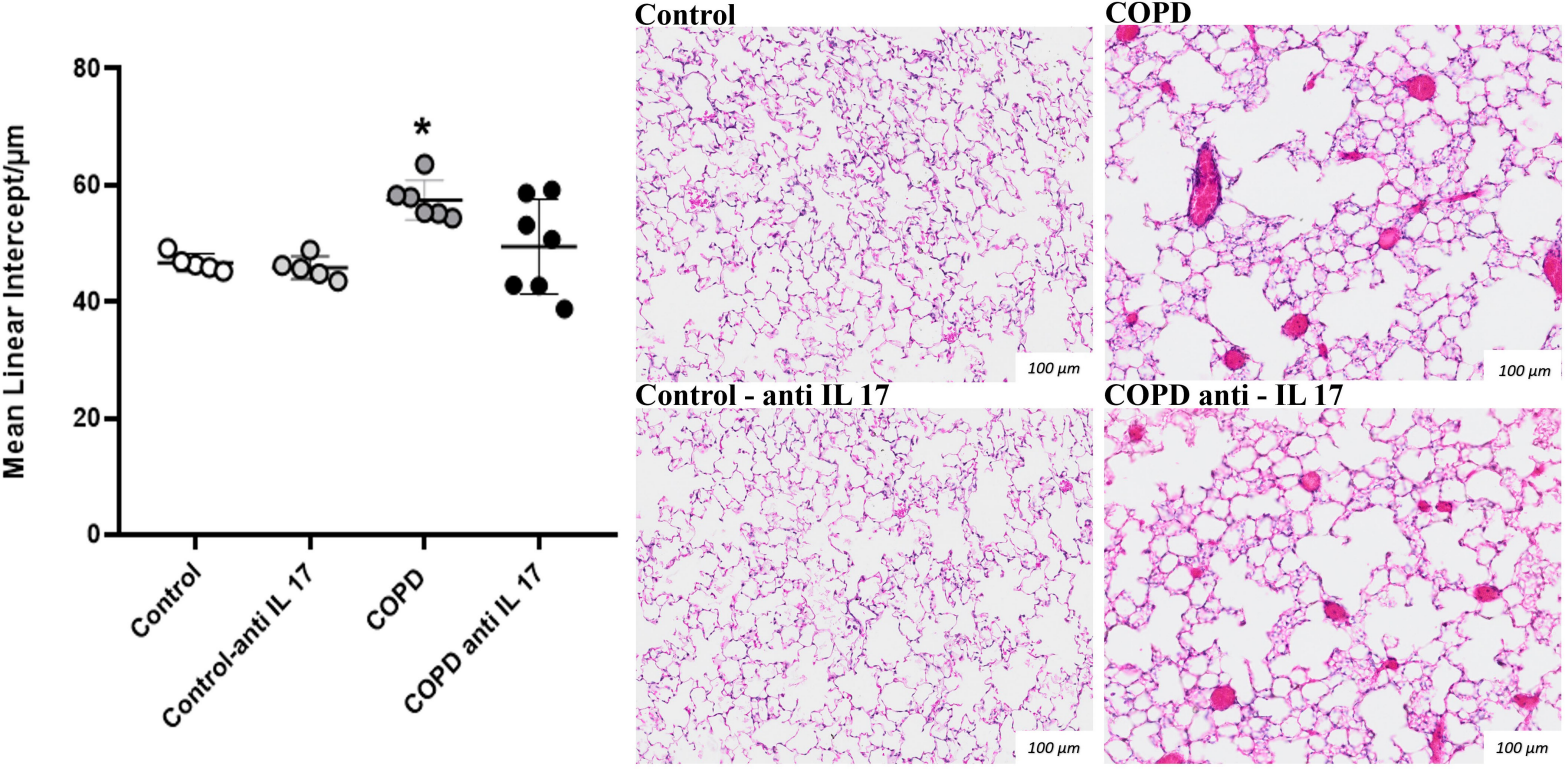
Mean linear intercept. Lm values in distal areas of the parenchyma (Control: *n* = 5; Control anti-IL-17: *n* = 5; COPD: *n* = 6; COPD anti-IL-17: *n* = 7). Data are expressed as mean ± SE. ^*^ *p* = 0.009 compared to the Control and COPD anti-IL-17 groups. Representative photomicrographs of Lm lung parenchyma areas are shown at × 100 magnification.

A significant increase in alveolar enlargement was observed in the COPD group compared to both the Control and anti-IL-17 COPD groups, as evidenced by mean linear intercept analysis (**Figure 1**). These results suggest that IL-17 blockade attenuates alveolar destruction typically seen in this experimental model.

### Decrease in parameters related to respiratory mechanics

**Figure 2:**
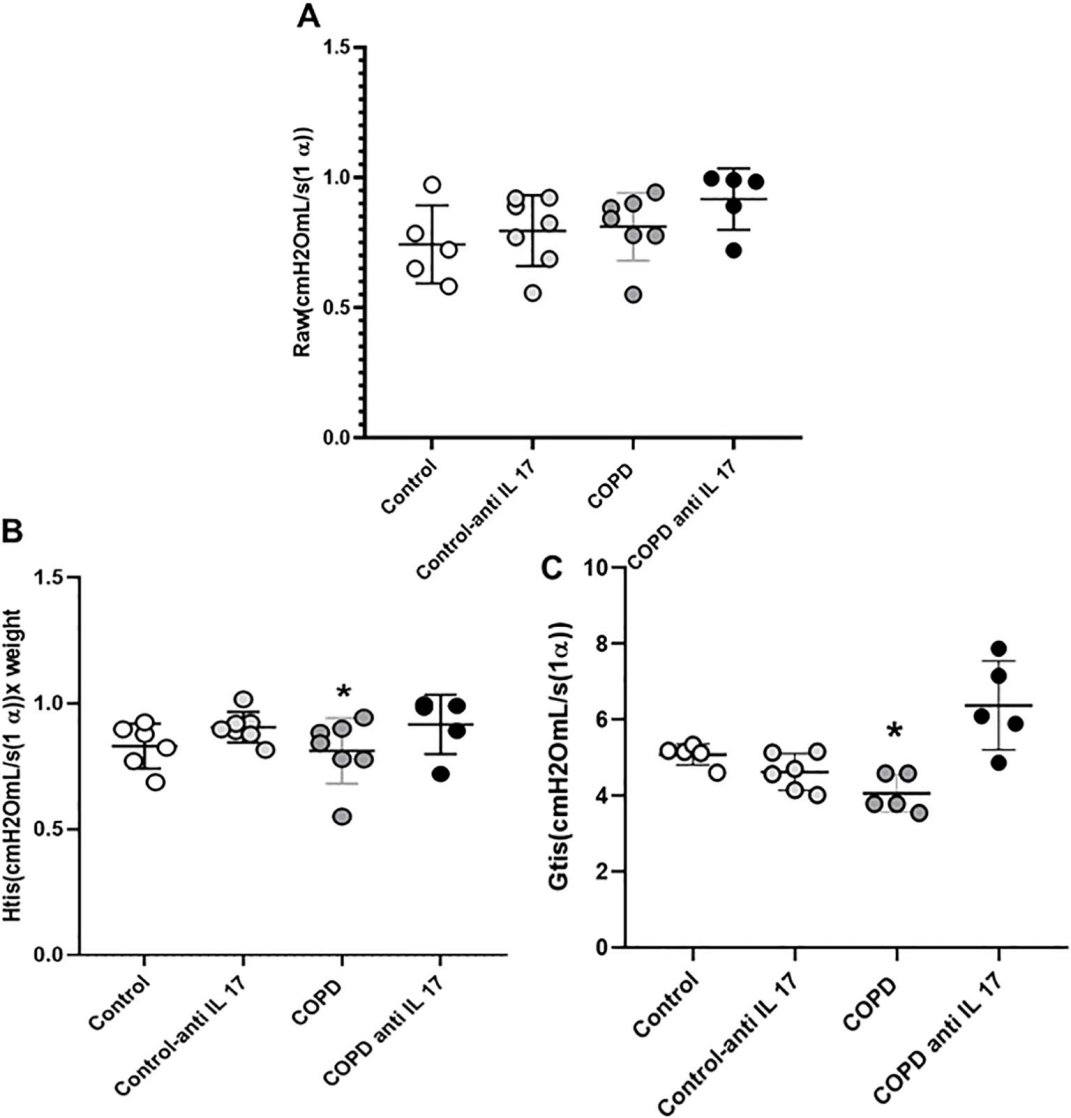
Respiratory mechanics. **(A)** Raw parameter (Control: *n* = 5; Control anti-IL-17: *n* = 7; COPD: *n* = 7; COPD anti-IL-17: *n* = 5). **(B)** Htis values (Control: *n* = 5; Control anti-IL-17: *n* = 7; COPD: *n* = 7; COPD anti-IL-17: *n* = 5). **(C)** Gtis values (Control: *n* = 5; Control anti-IL-17: *n* = 6; COPD: *n* = 5; COPD anti-IL-17: *n* = 5). Data are expressed as mean ± SE. ^*^ *p* ≤ 0.001 compared to other groups.

No statistically significant differences were observed in the RAW parameter (airway resistance) among the experimental groups (**Figure 2A**). A reduction in Htis values (tissue elastance) (**Figure 2B**) and in Gtis values (tissue resistance) (**Figure 2C**) was found in the COPD group compared to the other groups; however, treatment with anti-IL-17 reversed these changes in both parameters.

### Density of cells in the peribronchoalveolar space

**Figure 3:**
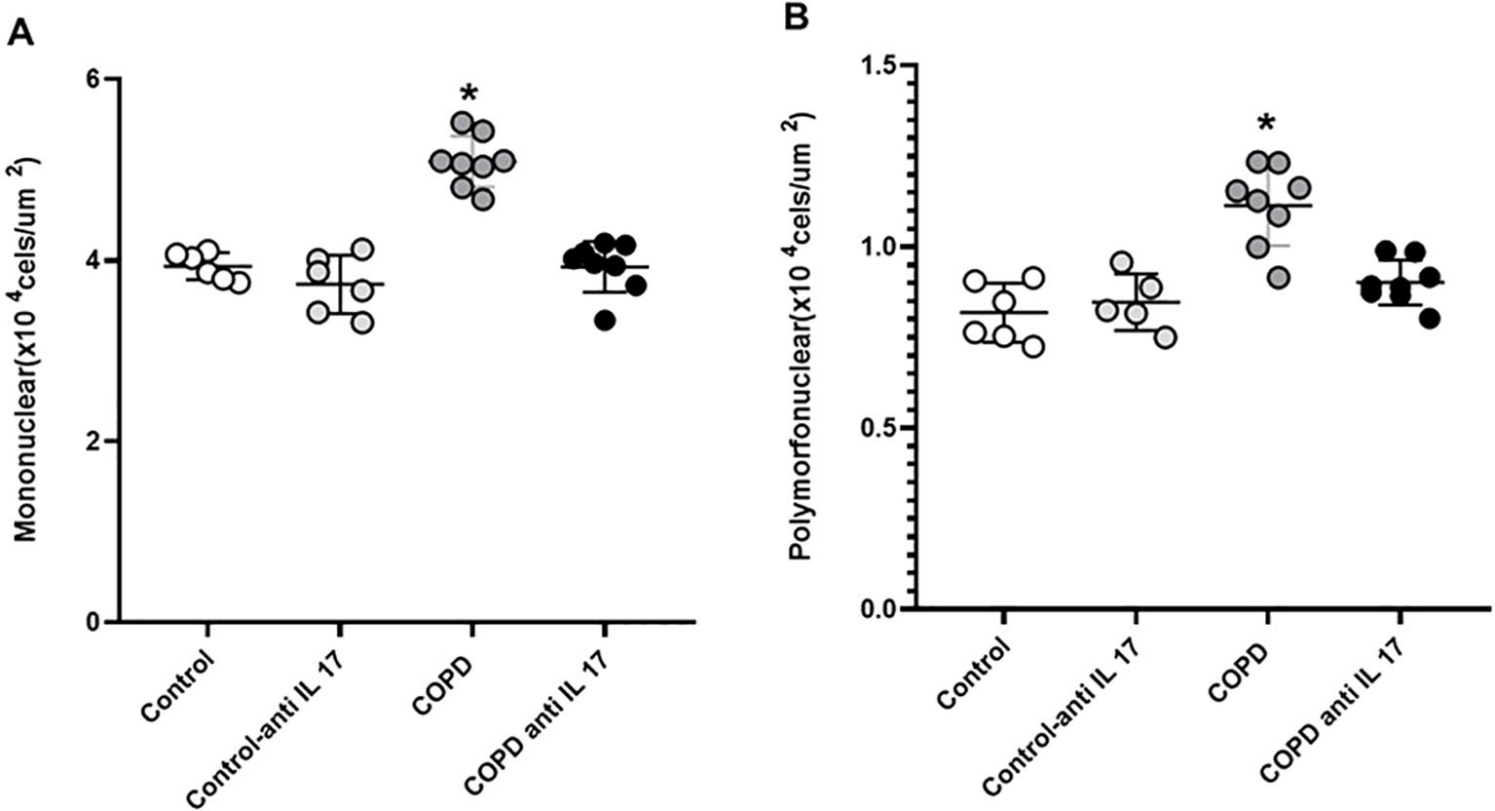
Mononuclear and polymorphonuclear cells. **(A)** Mononuclear cells. **(B)** Polymorphonuclear cells. Control: *n* = 6; Control anti-IL-17: *n* = 6; COPD: *n* = 8; COPD anti-IL-17: *n* = 8. Data are expressed as mean ± SE. ^*^ *p* ≤ 0.001 compared to the Control and COPD anti-IL-17 groups.

There was an increase in mononuclear and polymorphonuclear cells in the COPD group compared to the Control and COPD anti-IL-17 groups (**Figure 3**).

### Immunohistochemistry

**Figure 4:**
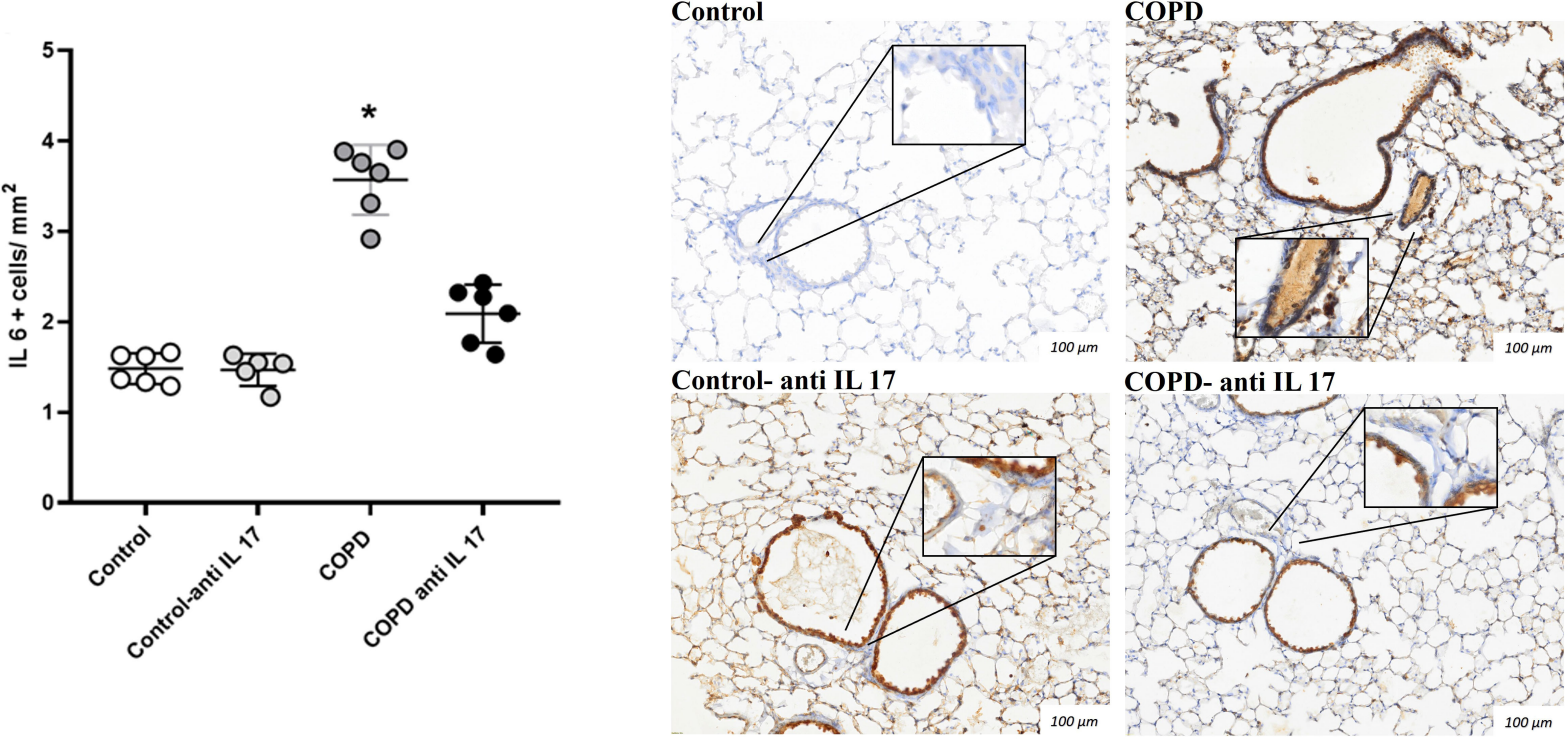
Immunohistochemistry by IL-6. Control: *n* = 6; Control anti-IL-17: *n* = 6; COPD: *n* = 6; COPD anti-IL-17: *n* = 6. Data are expressed as mean ± SE. ^*^ *p* ≤ 0.001 compared to the Control and COPD anti-IL-17 groups. Representative photomicrographs of IL-6^+^ cells in peribronchovascular areas are shown at × 100 magnification, with images at × 1,000 magnification presented in each inset.

**Figure 5:**
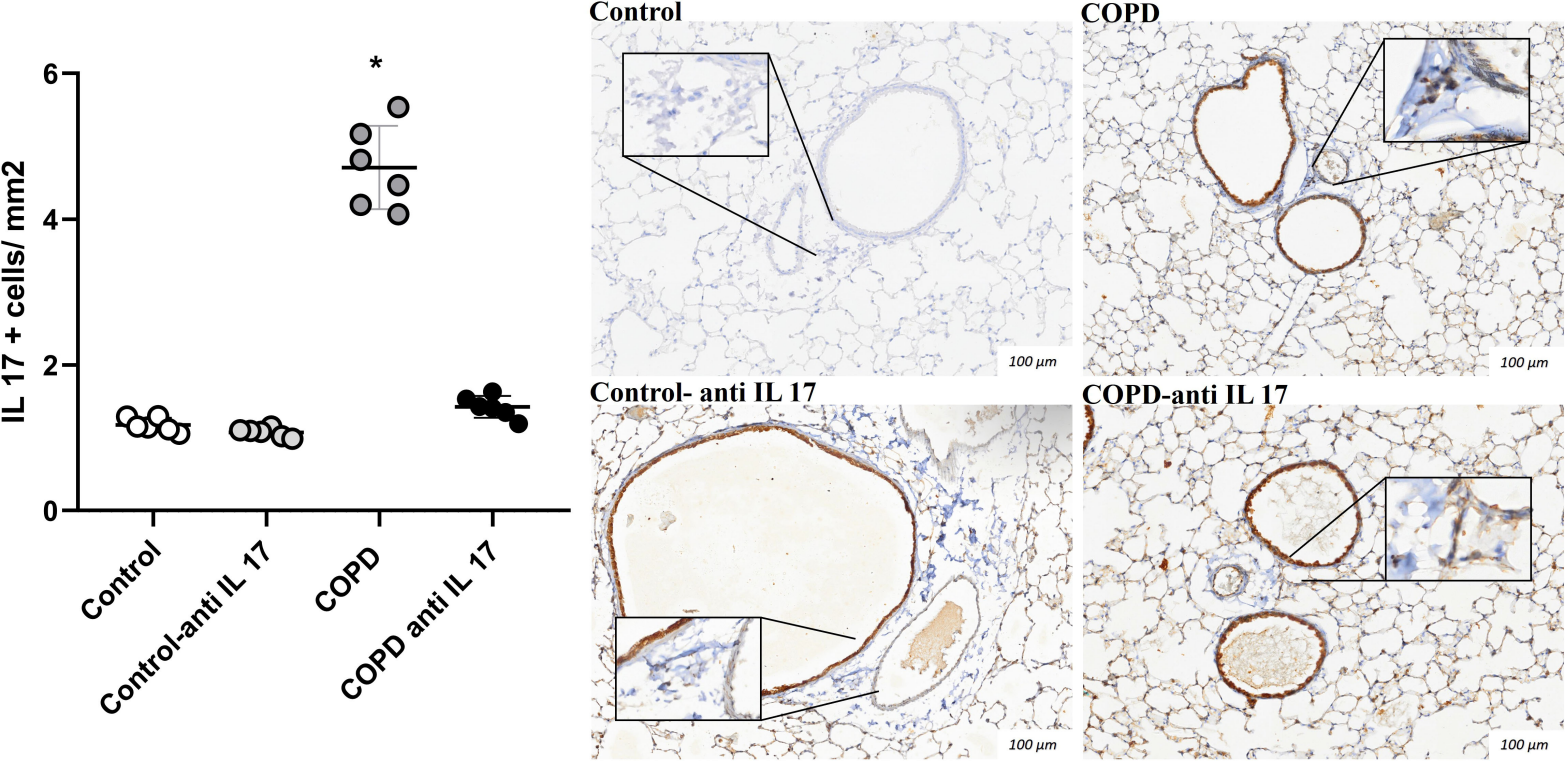
Immunohistochemistry by IL-17. Control: *n* = 6; Control anti-IL-17: *n* = 6; COPD: *n* = 6; COPD anti-IL-17: *n* = 6. Data are expressed as mean ± SE. ^*^ *p* ≤ 0.001 compared to the Control and COPD anti-IL-17groups. Representative photomicrographs of IL-17 cells in peribronchovascular areas are shown at × 100 magnification, with images at × 1,000 magnification presented in each inset.

There was an increase in IL-17-positive cells detected by immunohistochemistry compared to the expression in the Control and COPD anti-IL-17 groups (**Figures 4**, **5**).

### Increased expression of cytokines by ELISA

**Figure 6:**
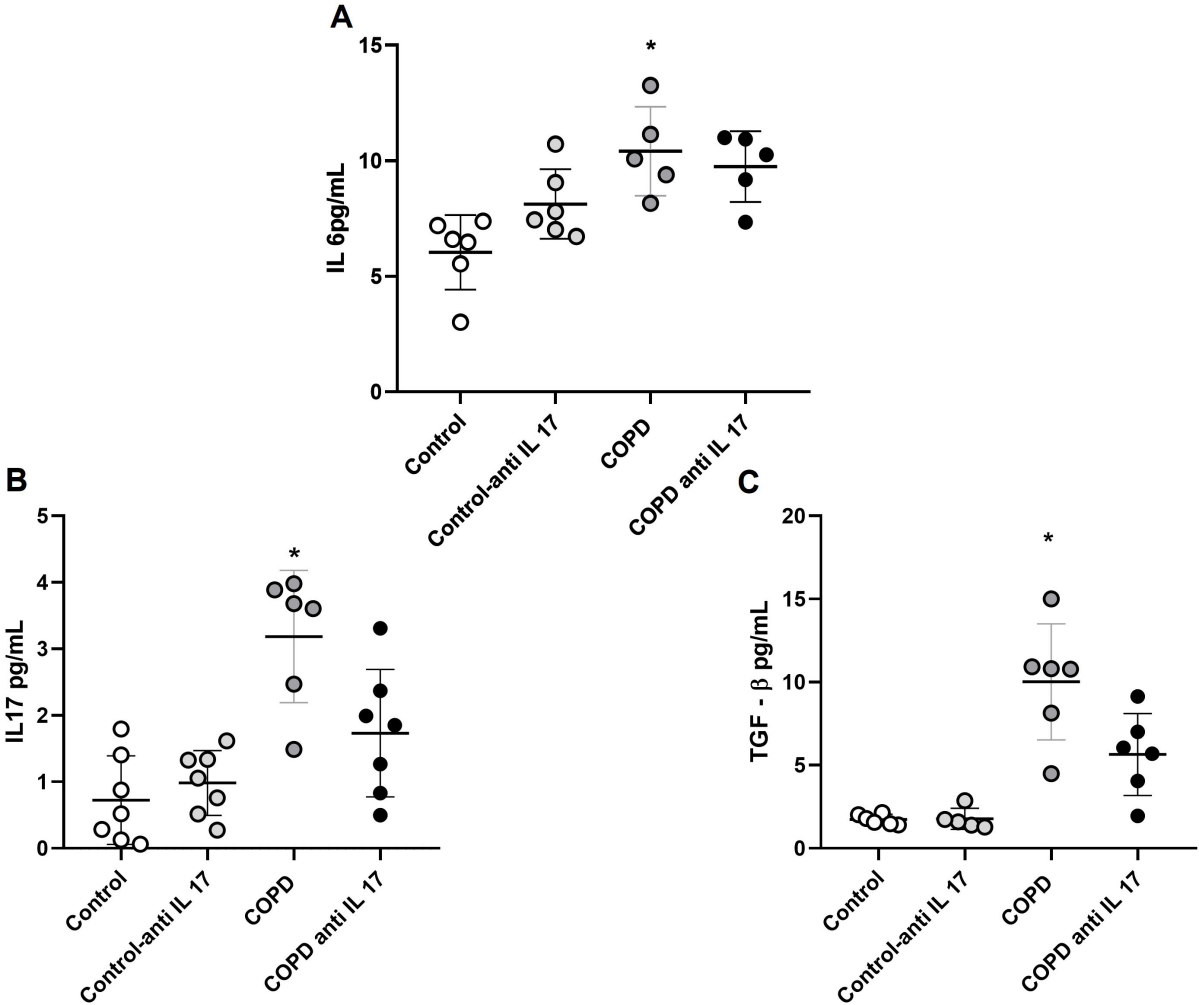
Expression of cytokines. **(A)** IL-6 protein levels (Control: *n* = 6; Control anti-IL-17: *n* = 6; COPD: *n* = 5; COPD anti-IL-17; *n* = 5). **(B)** IL-17 protein levels (Control: *n* = 7; Control anti-IL-17: *n* = 7; COPD: *n* = 6; COPD anti-IL-17; *n* = 7). **(C)** TGF-β protein levels (Control: *n* = 6; Control anti-IL 17: *n* = 6; COPD: *n* = 6; COPD anti-IL-17; *n* = 6). Data are expressed as mean ± SE. **(A)** ^*^ *p* = 0.002 compared to the Control group. **(B)** ^*^ *p* = 0.004 compared to all groups. **(C)** ^*^ *p* = 0.003 compared to all groups.

There was an increase in IL-6 protein levels in the groups exposed to cigarette smoke compared to the control group (**Figure 6**). Additionally, IL-17 and TGF-β protein levels were elevated in the group exposed to cigarette smoke compared to both the Control and COPD anti-IL-17 groups (**Figures 6B, C**).

### Gene expression

**Figure 7:**
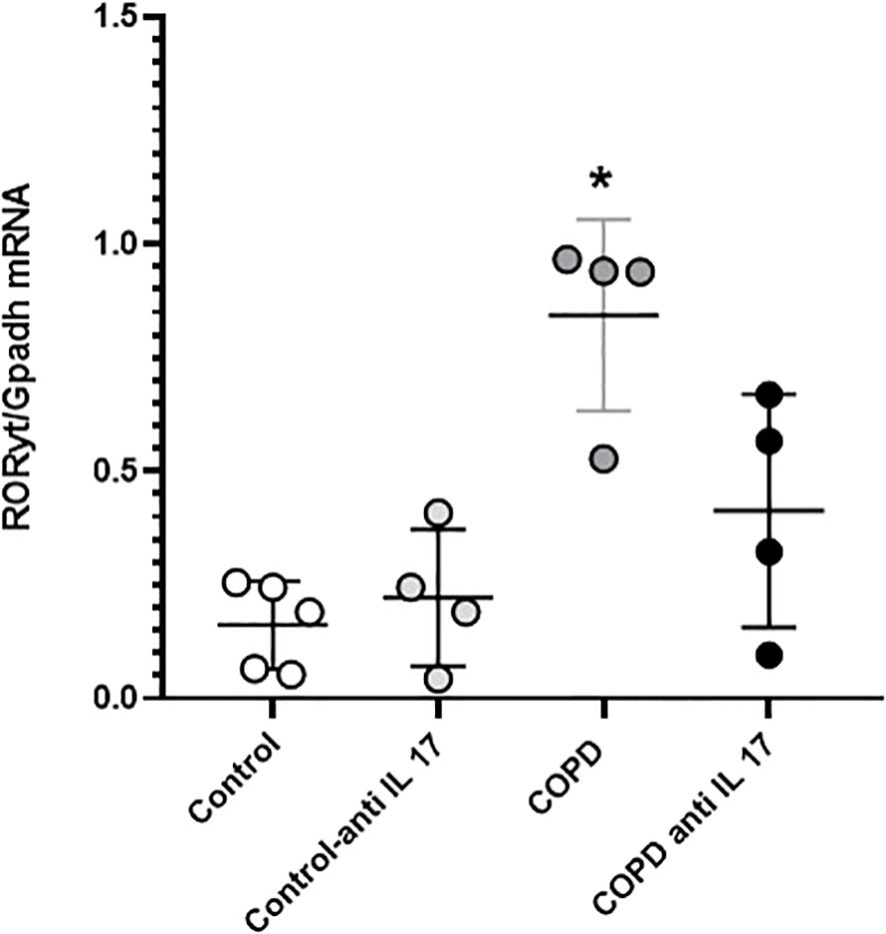
*RORγt* gene expression. Control: *n* = 5; Control anti-IL-17: *n* = 4; COPD: *n* = 4; COPD anti-IL-17; *n* = 4. Data are expressed as mean ± SE. ^*^ *p* = 0.001 compared to the Control group.

Gene expression assessment for *RORγt* showed an increase in cigarette smoke-exposed groups compared to the others (**Figure 7**).

## Discussion

COPD remains a major therapeutic challenge, as current treatment options are primarily aimed at relieving symptoms and reducing exacerbations, without effectively reversing tissue damage. In this context, identifying the molecular targets involved in disease progression is essential for developing more effective therapeutic strategies.

Our results showed that IL-17-neutralizing antibody administration decreases the inflammatory response mediated by Th17 cytokines in the lungs, mitigating the functional and structural changes induced by CS exposure in an experimental model of COPD.

We observed a recovery of lung elastic recoil through the analysis of Htis and Gtis after IL-17-neutralizing antibody administration in animals exposed to CS, which is consistent with findings related to the Lm parameter. The increased alveolar enlargement observed in the CS group was not present in animals exposed to CS that received IL-17 inhibitor treatment. The analysis of mononuclear and polymorphonuclear cells evaluated in the peribronchovascular areas revealed that the administration of the IL-17-neutralizing antibody inhibits the migration of these inflammatory cells to the lungs. Neutrophils and macrophages are recognized for their importance in alveolar parenchyma destruction by delivering matrix metalloproteinases (MMPs) in COPD (27). Previously, we have shown that CS exposure induced a microenvironmental stimulus related to M1- and M2-like macrophage phenotypes similar to those observed in COPD patients (28). MMPs, particularly MMP-12 and MMP-9, play critical roles in the extracellular matrix degradation and remodeling during pulmonary inflammatory processes in COPD. MMP-9, primarily produced by neutrophils, contributes to alveolar parenchymal destruction and regulates the migration of inflammatory cells to the lungs (29–31). MMP-12, secreted mainly by alveolar macrophages, also promotes matrix degradation and facilitates cell migration. Therefore, the migration inhibition of these inflammatory cells to the lungs prevents the perpetuation of the alveolar wall destruction, which corroborates with respiratory mechanics and Lm data.

The importance of Th17 response has been described as playing a pivotal role in the development and progression of COPD (32, 33). The differentiation of CD4+ T cells to TH17 cells depends on cytokines in the microenvironment, such as IL-6, due to its importance in the expression of the nuclear factor *RORγt* (34–38). Interestingly, we did not observe the effects of IL-17 inhibitor on this interleukin’s expression in the lungs, since both groups exposed to CS showed IL-6 expression increased compared with the Control group. These results are in accordance with the analysis of *RORγt* gene expression in the lung homogenates, as they showed an increased expression of this nuclear factor in the COPD and COPD anti-IL-17 groups compared with the Control group. In contrast, the results of IL-17 expression in the lung homogenates revealed the effects of this inhibitor in diminishing this interleukin’s expression. Moreover, we observed a statistically significant decrease in +IL-17 lymphocytes in peribronchovascular areas in the COPD anti-IL-17 group compared with the COPD group.

The anti-IL-17A monoclonal antibody (clone 50104) is designed to neutralize the activity of IL-17A. It specifically binds to IL-17A, preventing its interaction with the IL-17RA/IL-17RC receptor, thereby blocking proinflammatory signaling mediated by this cytokine. Thus, we believe that the administration of this neutralizing antibody did not prevent the differentiation of TH17 cells, considering that IL-6 levels, as well as *RORγt* gene expression, remained increased even after the treatment, but it neutralized the activity of IL-17, which could explain the impairment in functional and structural changes.

In this experimental model, we previously demonstrated that after 1 month of CS exposure, the alveolar walls enlarged, showing progression over time, and elastic recoil loss was observed from the third month (39). In the present study, IL-17 inhibitor administration begins only in the fifth month, after alveolar enlargement and changes in lung mechanics had already occurred. Nevertheless, we observed recovery of the lung parenchyma tissue and improvements in lung mechanics parameters. Further investigation is necessary to better understand the impact of this inhibitor administration on the remodeling of extracellular matrix components, mediated by macrophages and neutrophils through the release of metalloproteases and their inhibitors. This may help explain the recovery of alveolar parenchyma and the improvement in respiratory mechanics parameters, even after lung tissue destruction has been established.

In recent years, several monoclonal antibodies have been developed and tested as potent inhibitors of IL-17 in various inflammatory diseases to suppress its induction or eliminate IL-17-producing cells (40). In psoriasis, several anti-IL-17 monoclonal antibodies are currently used in treatment, targeting IL-17A or the IL-17RA receptor and blocking the inflammatory pathway involved in the pathogenesis of this disease (41, 42).

Beyond cutaneous involvement, psoriasis is increasingly recognized as a systemic inflammatory disorder associated with multiple comorbidities, including COPD. Epidemiological evidence supports a significantly increased risk of COPD among patients with psoriasis, with consistent findings across population-based cohorts and meta-analyses (15, 43–45).

Although the role of IL-17 released by innate and adaptive immune cells in the development and progression of COPD has been extensively studied, few investigations have assessed the effects of IL-17 inhibition once the disease is established, and even fewer have explored the long-term outcomes following treatment.

This is the first study to demonstrate the potential therapeutic effects of administering an IL-17-neutralizing antibody in restoring functional and structural changes in a COPD-induced model caused by CS exposure.

Our study has some limitations, as we did not evaluate how this treatment affects the lung tissue extracellular matrix components in relation to improvements in functional and structural parameters.

## Conclusions

In conclusion, this study demonstrates the benefits of administering IL-17-neutralizing antibodies in controlling the inflammatory response induced by CS exposure in mice, mitigating both functional and structural changes in a CS-induced COPD model. These findings underscore the central role of the IL-17 pathway in COPD progression, suggesting that targeting this cytokine may represent a promising therapeutic strategy. Further studies are needed to evaluate the long-term effects and the potential clinical applicability of this approach.

## Data Availability

The raw data supporting the conclusions of this article will be made available by the authors, without undue reservation.
